# Dietary total, plant and animal protein intake in relation to metabolic health status in overweight and obese adolescents

**DOI:** 10.1038/s41598-022-14433-1

**Published:** 2022-06-16

**Authors:** Keyhan Lotfi, Sobhan Mohammadi, Saeideh Mirzaei, Ali Asadi, Masoumeh Akhlaghi, Parvane Saneei

**Affiliations:** 1 Department of Community Nutrition, School of Nutritional Sciences and Dietetics, Tehran University of Medical Sciences, Tehran, Iran; 2 Department of Community Nutrition, School of Nutrition and Food Science, Food Security Research Center, Students’ Research Committee, Isfahan University of Medical Sciences, Isfahan, Iran; 3 Department of Community Nutrition, School of Nutrition and Food Sciences, Shiraz University of Medical Sciences, Shiraz, Iran; 4 Department of Exercise Physiology, School of Physical Education and Sport Sciences, University of Tehran, Tehran, Iran; 5 Department of Community Nutrition, School of Nutrition and Food Science, Food Security Research Center, Isfahan University of Medical Sciences, PO Box 81745-151, Isfahan, Iran

**Keywords:** Endocrine system and metabolic diseases, Metabolic disorders, Paediatric research, Nutrition, Paediatrics

## Abstract

Few studies have investigated dietary total protein intake and its subtypes in relation to metabolic health status. We explored the relation between dietary total, plant and animal protein intake with metabolic health status in Iranian overweight/obese adolescents. Overweight/obese adolescents (n = 203) were selected for this cross-sectional study by multistage cluster random-sampling method. A validated food frequency questionnaire was used to evaluate dietary intakes. Total, plant and animal protein intake were considered as percentage of energy intake. Anthropometric indices, blood pressure, lipid and glycemic profiles were collected. Participants were classified as metabolically healthy obese (MHO) or unhealthy obese (MUO) based on International Diabetes Federation (IDF) and IDF/Homeostasis Model Assessment Insulin Resistance (HOMA-IR) definitions. Subjects had a mean age of 13.98 years, and 50.2% of them were girls. Based on IDF criteria, adolescents in the top tertile of total (OR = 0.32; 95% CI 0.13–0.77), plant (OR = 0.30; 95% CI 0.10–0.91), and animal (OR = 0.20; 95% CI 0.08–0.54) protein intake had lower odds of being MUO compared to the reference category. Considering IDF/HOMA-IR criteria, subjects in the highest tertile of total (OR = 0.31; 95% CI 0.12–0.79) and animal (OR = 0.17; 95% CI 0.06–0.49) protein intake were less likely to be MUO. However, no substantial association was observed with plant protein intake. Also, an inverse association was observed between each SD increase in total and animal protein with MUO odds. We found inverse association between total, plant and animal protein intake and chance of being MUO in adolescents. Further prospective studies are needed to confirm the findings.

## Introduction

The growing prevalence of overweight and obesity among children and adolescents has become a global public health concern and imposed several health challenges to the societies^1,2^. Adolescence is a vital phase for the onset of obesity and its related chronic diseases such as cardiovascular disease and type 2 diabetes (T2D), that could persist into adulthood^3,4^. More than 330 million children and adolescents aged 5–19 were overweight or obese in 2016^5^. About 4 million of Iranian children and adolescents have been predicted to be overweight or obese by 2025^6^.

In the recent years, metabolic health status of overweight and obese individuals has been also gained great attention^7^. Metabolically healthy obese (MHO) subjects are those with normal cardio metabolic risk factors such as blood glucose, blood lipid and blood pressure^8^. On the other hand, individuals with abnormal metabolic status are considered as metabolically unhealthy obese (MUO) subjects^8^. In addition to genetic, lifestyle risk factors such as diet and physical activity might contribute to the presence of MHO and MUO^9^.

An increasing trend in dietary total energy intake has been indicated worldwide, and diets have been shifted from plant-based sources to calorie-dense foods^10^. Therefore, special consideration should be devoted to the composition of the diet, especially its macronutrient content, because they are participating in different metabolic pathways^11^. There is a lack of consensus on a single optimal distribution for macronutrients intake that could support lifelong health, and is greatly differed by the nations and food systems^12^. Among macronutrients, limited research has been focused on the relation between protein intake and metabolic status, especially among children and adolescents. Dietary protein is crucial for optimal growth of children and adolescents; however, excess amounts could be metabolized into fat^13^. An earlier study indicated a positive link between higher protein consumption in childhood and obesity later in life^14^. Conversely, a study among European adolescents found a protective association between plant protein intake and obesity, but no substantial relation was found for other cardio-metabolic risk factors^15^. On the other hand, dietary protein could have an insulin secretory effect and might results in decreased glucose levels^16^. However, a recent clinical trial found a sustained increase in blood glucose following a high protein diet^17^. Furthermore, a cross-sectional study among European adolescents found inverse association between total protein intake and diastolic blood pressure^18^. Another study in Iran has shown inverse associations between triglyceride and cholesterol levels and DASH diet, which has considerable amounts of plant protein^19^. It seems that the source of protein intake is also an important factor and should be acknowledged^20^. Based on a cohort study, higher consumption of animal-based proteins might increase the risk of hypertension and obesity^20^. Whereas, favorable effects of plant protein on metabolic factors have been suggested^21^. Furthermore, it should be noted that in an isoenergetic condition, higher intake of a macronutrient is always accompanied by lower intake of another macronutrient. As far as we know, limited data have evaluated the association between dietary total protein intake and its subtypes with metabolic health status of overweight or obese adolescents in the Middle East countries, where carbohydrates are the predominant source of energy intake. Furthermore, previous studies have not considered the substitution effect of carbohydrate by protein on metabolic health. Therefore, the current study was conducted to explore the association between dietary total, plant, and animal protein intake with healthy/unhealthy phenotypes among a sample of Iranian overweight/obesity adolescents.

## Methods and materials

### Participants

In the present cross-sectional study, 203 adolescents (102 girls and 101 boys) aged 12 to < 18 years old were enrolled. The sample size of the current investigation was calculated according to earlier studies, that have shown about 60% of overweight and obese adolescents suffer from MUO^22,23^. Therefore, the minimum required sample size was calculated to be 188 adolescents, considering type I error of 0.05, a power of 80%, desired confidence interval of 95%, and a precision (d) of 7%. Therefore, 203 overweight/obese adolescents were recruited for our study. A multi-level cluster random-sampling approach was applied to select adolescents from sixteen schools in five major regions of Isfahan, Iran. At first, adolescents’ body weight and height were measured, and their body mass index (BMI) was computed using the Quetelet formula [weight (kg)/height^2^ (m)]. Then, according to the World Health Organization growth curve age-sex specific BMI percentiles^24^, subjects were classified as normal-weight, overweight, or obese. Overweight and obese adolescents were considered to participate in the current investigation. Individuals with various socioeconomic statuses were included in our study. Adolescents who used any medication or supplement that might affect body weight, blood pressure, blood glucose, or lipid profiles as well as those who were on a weight-loss diet were not eligible for the present analysis. We additionally excluded individuals with genetic or endocrine disorders (Cushing's syndrome, type 1 diabetes and hypothyroidism). Informed consent forms were taken from all participants and their parents. Written informed agreement was obtained from each participant. In addition, informed consents were obtained from their parents as few minors were involved in the study. The study protocol was approved by the local Ethics Committee of Isfahan University of Medical Sciences.

### Dietary intake assessment

Dietary intakes of individuals in the preceding year was obtained by a validated 147-items food frequency questionnaire (FFQ)^25^. Previous studies on Iranian adolescents revealed that this FFQ could be an accurate indicator of dietary intakes in relation to various diseases including metabolic syndrome and obesity phenotypes^19,26–28^. This questionnaire asked consumption of each food item on a daily, weekly, or monthly frequency and the portion size of consumption. Reported portion sizes and frequencies were multiplied, and then converted to grams per day using household measurements^29^. Nutritionist IV software was applied to assess energy and nutrients intake. Nutritionist IV used the USDA's food composition database. This approach has been previously suggested to be reasonable for the countries that have not developed a comprehensive food composition table, because most of the foods’ nutrients (e.g. an apple) are not much different among regions^30,31^. For some specific Iranian foods that do not exist in an American diet, we have chosen the most similar item from USDA database.

The amounts (grams per day) of protein contributed to plant-based foods such as vegetables, grains, nuts, and legumes were summed up to calculate dietary plant protein intake. Same method was applied to calculate dietary animal protein intake, considering animal-based foods such as meats and meat products, dairy, egg, and fish. Dietary total protein intake was equal to the sum of plant and animal protein intake.

### Assessment of anthropometric indices and cardio metabolic risk factors

Barefoot standing height was measured by two trained dietitians to the nearest 0.1 cm using a stadiometer. Adolescents’ weight was also assessed by a calibrated electronic scale to the nearest 0.1 kg without shoes and in minimal clothes. Then, defined World Health Organization growth curve age-sex specific BMI percentiles were used to categorize adolescents as overweight (85th < BMI < 95th percentile) or obese (BMI > 95th percentile)^24^. A flexible tape was used to measure waist circumference (WC) to the nearest 0.1 cm after normal respiration and with no pressure on the body surface. To assess blood pressure, subjects were first asked to sit in a chair with back supported and feet flat on floor, and stay relax without moving or talking for 5 min^32^. Also, they were asked not to talk or move during the measurement. Systolic blood pressure (SBP) and diastolic blood pressure (DBP) were measured for two times (after a 15-min recovery time) by using a mercury sphygmomanometer with an appropriate cuff size, and the average value was considered in our analysis. Fasting blood glucose (FBG), triglycerides (TG), insulin and high density lipoprotein cholesterol (HDL-c) levels were assessed using fasting blood samples. According to Homeostasis Model Insulin Resistance (HOMA-IR) formula^33^, we calculated Insulin resistance (IR) for each participant: [Fasting glucose (mg/dL) × fasting insulin (μU/mL)]/405.

### Assessment of metabolic status

Two different methods were applied to determine MHO and MUO adolescents. First method was based on International Diabetes Federation (IDF) criteria^34^ that includes the following risk factors: (1) elevated blood pressure (≥ 130/85 mmHg), (2) increased levels of FBG (≥ 100 mg/dL), (3) elevated TG levels (≥ 150 mg/dL), and (4) low HDL-c (< 40 mg/dL for subjects < 16 years, < 50 mg/dL for ≥ 16 years girls, and < 40 mg/dL for ≥ 16 years boys). Participants were defined as MUO if they had at least two of the mentioned risk factors. Otherwise, they classified as MHO individuals. Second method considered the presence of IR in addition to IDF criteria. By this approach, adolescents with HOMA-IR scores ≥ 3.16^35,36^, and ≥ 2 of the above-mentioned risk factors were identified as MUO. Individuals with HOMA-IR values < 3.16 were determined as MHO, even if they had ≥ 2 risk factors.

### Assessment of other variables

Information about adolescents’ age, gender, medical history, medications and supplements usage was collected through a questionnaire. Socioeconomic status (SES) of subjects was evaluated by a validated demographic questionnaire^37^. The following items were considered in this questionnaire: family size, parental education level, parental job, having personal room, taking trips in a year, having cars in the family, and having computers/laptops. Finally, the SES was calculated to have a total score. Physical Activity Questionnaire for Adolescents (PAQ-A) was applied to gather data on individuals’ physical activity level^38^. This is a 9-item questionnaire estimating physical activity level in the previous week. The first 8 items of PAQ-A were 5-point rating questions; a score of 1 indicated the lowest and 5 showed the highest level of physical activity. The last question assessed unusual activity in the last week. The first 8 items asked about (1) spare time activities (i.e. soccer, jogging, swimming, walking for exercise and etc.); their activity during (2) physical education classes, (3) lunch, (4) right after school, (5) evenings, and (6) the last weekend; (7) how they describe themselves; and (8) the levels of activity in each day. The average score of all week days was considered for the last item. Finally, the sum of the scores classified participants as sedentary or not having an orderly week activity (score < 2), less active (3 < score ≤ 2), active (score ≥ 3), and very active (score ≥ 4). Since a small number of participants were categorized as sedentary and very active, we combined sedentary with less active and active with very active levels to have two final categories (low and high levels of physical activity).

### Statistical analysis

First, dietary total, plant and animal intakes were considered as a percentage of total energy intake. Then, study participants were classified based on tertiles of dietary total (T_1_: 9.4–13.3, T_2_: 13.4–14.9, T_3_:14.9–21.2% of energy intake), plant (T_1_: 4.1–6.8, T_2_: 6.8–7.6, T_3_:7.6–13.4% of energy intake), and animal (T_1_: 2.4–6.1, T_2_: 6.1–7.8, T_3_:7.8–12.9% of energy intake) protein intake. General characteristics of the study subjects were reported as mean and standard deviation (SD) for continuous and percentage for categorical variables. The differences of participants’ general characteristics across tertiles of dietary total protein intake were evaluated through Analysis of Variance (ANOVA) and Chi-square test for continuous and categorical variables, respectively. Analysis of Covariance (ANCOVA) was applied to assess individuals’ dietary intakes across tertiles of total protein intake. In ANCOVA, total energy intake and macronutrients were adjusted for age and gender. Other dietary values were adjusted for age, gender, and energy intake. The relation between dietary total, plant, and animal protein intake and chance of MUO was assessed through binary logistic regression in crude and multivariable-adjusted models. In all analyses, the first tertile was considered as the reference category. In the first model, age (continuous), gender (girl/boy) and total energy intake (continuous) were taken into account as covariates. In the second model further adjustments were done for SES (low/moderate/high) and physical activity level (low/high). In the third model, dietary total fat intake (percentage of energy), plant protein intake (percentage of energy) (for animal protein), animal protein intake (percentage of energy) (for plant protein), and BMI (continuous) were additionally considered. We used leave-one-out model^39,40^ to assess the isocaloric substitution of carbohydrate with total protein intake by including total energy and fat intake in the model to keep them constant, while leaving carbohydrate intake out of the model. The obtained regression coefficient for total protein intake was the log odds ratio for substituting total protein for an isocaloric amount of carbohydrate. Same approach was considered in case of plant and animal protein intake. Tertiles of total protein intake and its subtypes were considered as an ordinal variable to assess the trend of odds ratios. We have additionally done the same analyses for each 1 SD increase in total, plant and animal protein intake as an independent variable. All statistical analyses were performed by SPSS software (version 20; SPSS Inc, Chicago IL). P_value_ < 0.05 was considered as the level of significance.

### Ethical approval

The study procedure was performed according to declaration of Helsinki and STROBE checklist. All participants provided informed written consent. The study protocol was approved by the local Ethics Committee of Isfahan University of Medical Sciences.

### Consent to participate

Informed consent was obtained from all participants involved in the study.

## Results

In total, 203 adolescents with a mean age of 14.0 ± 1.6 years were included in our study; 50.2% of them were girls. Based on IDF definition, 79 subjects were categorized as MUO individuals, while 67 adolescents were MUO considering IDF/HOMA-IR criteria. Table 1 reports general characteristics of adolescents across tertiles of dietary total protein intake. Participants in the top tertile of dietary total protein intake had higher physical activity level, FBG, insulin, HOMA-IR, triglycerides, and HDL cholesterol levels compared to the bottom tertile. However, differences in age, weight, BMI, gender, SES, SBP and DBP were not substantial across tertiles of dietary total protein intake.

**Table 1:** General characteristics of study participants across tertiles of dietary total protein intake (n = 203)^1^.

	Tertiles of dietary total protein intake			P^2^
	T_1_ (n = 67)	T_2_ (n = 68)	T_3_ (n = 68)	
Age (y)	13.9 ± 1.5	14.0 ± 1.7	14.0 ± 1.6	0.78
Weight (kg)	74.0 ± 11.7	72.9 ± 11.7	73.6 ± 11.6	0.86
Body mass index (kg/m^2^)	27.6 ± 2.9	27.3 ± 3.2	27.2 ± 3.6	0.71
**Gender (%)**				0.83
Boy	52.2	47.1	50.0	
Girl	47.8	52.9	50.0	
**Physical activity levels (%)**				< 0.001
Low	70.1	44.1	36.8	
High	29.9	55.9	63.2	
**Socioeconomic status levels (%)**				0.14
Low	29.9	38.2	19.1	
Moderate	40.3	39.7	52.9	
High	29.9	22.1	27.9	
Systolic blood pressure (mmHg)	116.8 ± 17.5	109.8 ± 21.0	111.7 ± 15.7	0.07
Diastolic blood pressure (mmHg)	74.4 ± 13.1	73.2 ± 10.5	72.9 ± 10.4	0.70
Fasting blood glucose (mg/dL)	100.8 ± 8.4	97.7 ± 9.3	96.0 ± 7.1	0.004
Insulin (µUI/mL)	25.5 ± 15.2	18.0 ± 9.2	17.8 ± 11.5	< 0.001
HOMA-IR index	6.3 ± 3.7	4.4 ± 2.4	4.3 ± 3.2	< 0.001
Triglycerides (mg/dL)	144.3 ± 68.3	110.1 ± 51.4	111.8 ± 73.2	0.003
HDL cholesterol (mg/dL)	42.2 ± 8.2	45.9 ± 6.5	46.3 ± 8.3	0.004

^1^All values are means ± standard deviation (SD), unless indicated.

^2^Obtained from ANOVA for continuous variables and chi-square test for categorical variables.

**Figure 1:**
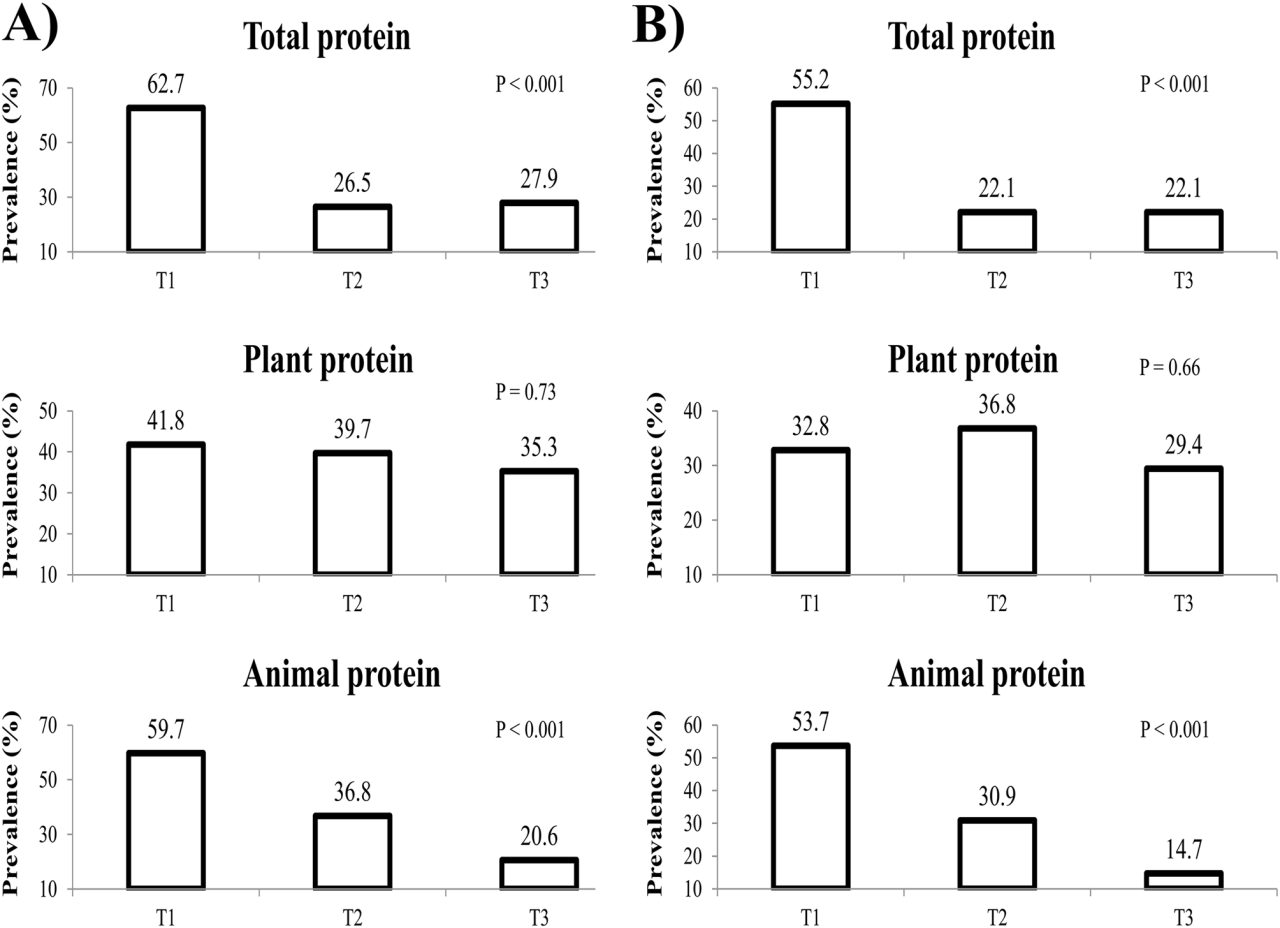
Prevalence of MUO across tertiles of dietary total, plant, and animal protein intake in the study population. (**A**) MUO based on IDF definition. (**B**) MUO based on IDF/HOMA-IR definition.

Figure 1 illustrates the prevalence of MUO in our study across dietary total, plant and animal protein intake tertiles. Considering IDF criteria, subjects had lower prevalence of MUO in the last tertile of dietary total (27.9 vs. 62.7%; P < 0.001), and animal protein intake (20.6 vs. 59.7%; P < 0.001), compared to the first tertile. According to IDF/HOMA-IR definition, adolescents had lower prevalence of MUO in the top tertile of dietary total (22.1 vs. 55.2%; P < 0.001), and animal protein intake (14.7 vs. 53.7%; P < 0.001). However, there were not significant differences in the prevalence of MUO across tertiles of dietary plant protein intake considering IDF (P = 0.73) or IDF/HOMA-IR (P = 0.66) criteria.

Mean (± SD) consumption of dietary total, plant and animal protein was 14.3 (± 2.0), 7.3 (± 1.6), and 7.0 (± 2.0) percent of the total energy intake, respectively. Adolescents’ dietary intakes of selected nutrients and food groups across tertiles of dietary total protein intake are shown in Table 2. Participants in the top tertile of total protein intake, compared to those in the first tertile, had a higher consumption of plant protein, animal protein, saturated fatty acids, fiber, red meats, vegetables, fruits, nuts, legumes and dairy. However, carbohydrates and grains intake was lower among the individuals in the highest tertile of total protein intake compared to the lowest one. Total energy and total fat intake were not substantially different between participants in the top and the bottom tertiles of total protein intake.

**Table 2:** Multivariable-adjusted intakes of selected food groups and nutrients of study participants across tertiles of dietary total protein intake (n = 203)^1^.

	Tertiles of dietary total protein intake			P^2^
	T_1_ (n = 67)	T_2_ (n = 68)	T_3_ (n = 68)	
Energy (kcal/d)	2899.9 ± 66.5	2893.3 ± 66.0	2856.5 ± 66.0	0.88
**Nutrients**				
Total protein (% of energy)	12.2 ± 0.12	14.2 ± 0.12	16.5 ± 0.12	< 0.001
Plant protein (% of energy)	6.9 ± 0.19	7.1 ± 0.18	8.0 ± 0.18	< 0.001
Animal protein (% of energy)	5.3 ± 0.18	7.1 ± 0.18	8.5 ± 0.18	< 0.001
Carbohydrates (% of energy)	59.9 ± 0.61	58.6 ± 0.61	56.4 ± 0.61	< 0.001
Fats (% of energy)	29.1 ± 0.64	28.6 ± 0.63	28.8 ± 0.63	0.84
Saturated Fatty acids (g/d)	25.9 ± 0.70	27.1 ± 0.70	29.0 ± 0.70	0.01
Dietary fiber (g/d)	17.3 ± 0.56	19.8 ± 0.56	21.2 ± 0.56	< 0.001
**Food groups (g/d)**				
Red meats	55.9 ± 3.9	72.3 ± 3.8	77.7 ± 3.8	< 0.001
Vegetables	202.1 ± 20.1	291.7 ± 20.0	333.3 ± 20.0	< 0.001
Fruits	287.3 ± 19.7	349.0 ± 19.5	360.6 ± 19.5	0.02
Grains	713.2 ± 16.5	678.8 ± 16.4	576.8 ± 16.4	< 0.001
Nuts	9.2 ± 1.3	12.1 ± 1.3	15.1 ± 1.3	0.01
Legumes	34.9 ± 3.2	44.5 ± 3.2	67.1 ± 3.2	< 0.001
Dairy	377.4 ± 22.8	510.6 ± 22.7	656.7 ± 22.7	< 0.001

^1^All values are means ± standard error (SE); energy and macronutrients intake were adjusted for age and gender; all other values and saturated fatty acids are adjusted for age, gender and energy intake.

^2^Obtained from ANCOVA.

**Figure 2:**
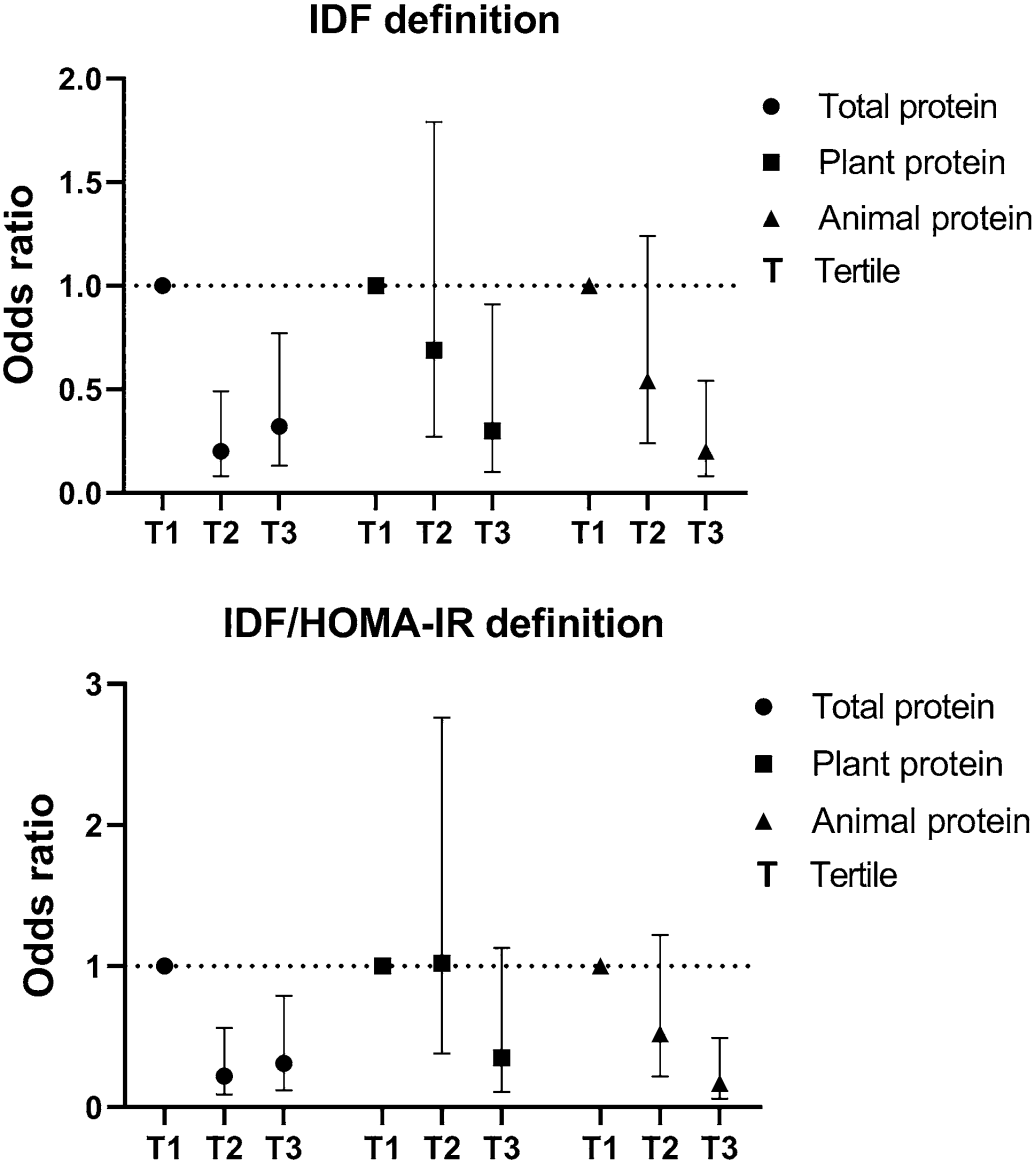
Multivariable-adjusted odds ratio and 95% CIs for MUO across tertiles of dietary total, plant, and animal protein intake. The estimates were adjusted for age, gender, energy intake, physical activity levels, socioeconomic status, total dietary fat intake, plant protein (for animal protein), animal protein (for plant protein) and BMI.

Table 3 and Fig. 2 provide crude and multivariable-adjusted odds ratios for MUO across tertiles of total, plant, and animal protein intake considering IDF criteria. Individuals in the top tertile of dietary total protein intake had lower odds of being MUO in crude (OR 0.23; 95% CI 0.11–0.48) and maximally-adjusted (OR 0.32; 95% CI 0.13–0.77) models compared to those in the bottom category. In the case of plant protein intake, there was not a significant association between the top tertile of dietary intake and possibility of being MUO (OR for T3 vs. T1: 0.76; 95% CI 0.38–1.52) in the crude model. However, after controlling all of the possible confounders this association became stronger and statistically significant (OR 0.30; 95% CI 0.10–0.91). Furthermore, participants in the last tertile of animal protein intake had lower odds of MUO in crude (OR for T3 vs. T1: 0.18; 95% CI 0.08–0.38) and fully-adjusted (OR for T3 vs. T1: 0.20; 95% CI 0.08–0.54) models. A significant trend was observed for MUO across tertiles of dietary total, plant, and animal protein intake after considering all confounders. Furthermore, each SD increase in dietary total and animal protein intake was respectively associated with 31%, and 37% reduced odds of being MUO in the fully-adjusted model. However, no significant linear association was seen between dietary plant protein intake and chance of being MUO.

**Table 3:** Multivariable-adjusted odds ratio for MUO (based on IDF criteria) across tertiles of dietary total, plant, and animal protein intake (n = 203)^1^.

	Tertiles of dietary protein intake				Per 1 SD increase
	T_1_	T_2_	T_3_	P_trend_	
**Total protein**					
Median (% of energy)	12.5	14.2	16.3		2.0
Participants/cases (n)	67/42	68/18	68/19		
Crude	1.00	0.21 (0.10–0.45)	0.23 (0.11–0.48)	< 0.001	0.58 (0.42–0.80)
Model 1	1.00	0.18 (0.08–0.39)	0.24 (0.11–0.51)	< 0.001	0.58 (0.41–0.81)
Model 2	1.00	0.20 (0.08–0.49)	0.32 (0.13–0.77)	0.01	0.69 (0.48–0.98)
Model 3	1.00	0.20 (0.08–0.49)	0.32 (0.13–0.77)	0.01	0.69 (0.48–0.99)
**Plant protein**					
Median (% of energy)	6.1	7.1	8.6		1.6
Participants/cases (n)	67/28	68/27	68/24		
Crude	1.00	0.92 (0.46–1.82)	0.76 (0.38–1.52)	0.44	1.01 (0.76–1.34)
Model 1	1.00	0.89 (0.43–1.83)	0.57 (0.27–1.19)	0.14	0.93 (0.69–1.26)
Model 2	1.00	0.93 (0.41–2.10)	0.52 (0.22–1.20)	0.14	0.92 (0.66–1.30)
Model 3	1.00	0.69 (0.27–1.79)	0.30 (0.10–0.91)	0.03	0.85 (0.57–1.26)
**Animal protein**					
Median (% of energy)	5.1	6.7	9.2		2.0
Participants/cases (n)	67/40	68/25	68/14		
Crude	1.00	0.39 (0.20–0.79)	0.18 (0.08–0.38)	< 0.001	0.57 (0.42–0.79)
Model 1	1.00	0.42 (0.20–0.85)	0.19 (0.08–0.42)	< 0.001	0.60 (0.43–0.84)
Model 2	1.00	0.61 (0.27–1.35)	0.28 (0.11–0.68)	0.01	0.72 (0.50–1.04)
Model 3	1.00	0.54 (0.24–1.24)	0.20 (0.08–0.54)	0.001	0.63 (0.42–0.95)

^1^All values are odds ratios and 95% confidence intervals. Model 1: Adjusted for age, gender, energy intake. Model 2: More adjustments for physical activity levels, socioeconomic status. Model 3: Further adjustments for total dietary fat intake, plant protein (for animal protein), animal protein (for plant protein) and BMI.

Crude and multivariable-adjusted odds ratios for MUO (based on IDF/HOMA-IR definition) across tertiles of total, plant, and animal protein intake are presented in Table 4 and Fig. 2. Adolescents in the third tertile of dietary total protein intake were less likely to be MUO in the crude model (OR 0.23; 95% CI 0.11–0.49) compared to the first tertile. This association remained significant after adjusting all of the potential confounders (OR 0.31; 95% CI 0.12–0.79). However, we did not find a significant relation between dietary plant protein intake and odds of being MUO in the crude (OR 0.85; 95% CI 0.41–1.77) and fully-adjusted (OR 0.35; 95% CI 0.11–1.13) models. Furthermore, dietary animal protein intake was significantly associated with reduced odds of being MUO in the crude (OR 0.15; 95% CI 0.07–0.34) and maximally-adjusted (OR 0.17; 95% CI 0.06–0.49) models. We found a significant trend for MUO across tertiles of dietary total and animal protein intake, but not for plant protein intake. Total and animal protein intake were related to 32% and 40% reduced odds of being MUO per 1 SD increase, however, no significant linear relation was seen between plant protein intake and possibility of being MUO.

**Table 4:** Multivariable-adjusted odds ratio for MUO (based on IDF/HOMA-IR criteria) across tertiles of dietary total, plant, and animal protein intake (n = 203)^1^.

	Tertiles of dietary protein intake				Per 1 SD increase
	T_1_	T_2_	T_3_	P_trend_	
**Total protein**					
Median (% of energy)	12.5	14.2	16.3		2.0
Participants/cases (n)	67/37	68/15	68/15		
Crude	1.00	0.23 (0.11–0.49)	0.23 (0.11–0.49)	< 0.001	0.58 (0.42–0.81)
Model 1	1.00	0.19 (0.08–0.43)	0.24 (0.11–0.53)	< 0.001	0.58 (0.41–0.82)
Model 2	1.00	0.23 (0.09–0.57)	0.33 (0.13–0.81)	0.01	0.69 (0.47–1.00)
Model 3	1.00	0.22 (0.09–0.56)	0.31 (0.12–0.79)	0.01	0.68 (0.46–0.99)
**Plant protein**					
Median (% of energy)	6.1	7.1	8.6		1.6
Participants/cases (n)	67/22	68/25	68/20		
Crude	1.00	1.19 (0.59–2.42)	0.85 (0.41–1.77)	0.67	1.04 (0.78–1.40)
Model 1	1.00	1.15 (0.54–2.44)	0.63 (0.28–1.38)	0.25	0.97 (0.71–1.34)
Model 2	1.00	1.27 (0.54–2.99)	0.58 (0.24–1.42)	0.25	0.96 (0.67–1.37)
Model 3	1.00	1.02 (0.38–2.76)	0.35 (0.11–1.13)	0.06	0.89 (0.58–1.34)
**Animal protein**					
Median (% of energy)	5.1	6.7	9.2		2.0
Participants/cases (n)	67/36	68/21	68/10		
Crude	1.00	0.39 (0.19–0.78)	0.15 (0.07–0.34)	< 0.001	0.55 (0.40–0.77)
Model 1	1.00	0.41 (0.20–0.86)	0.16 (0.07–0.38)	< 0.001	0.58 (0.41–0.83)
Model 2	1.00	0.59 (0.26–1.33)	0.23 (0.09–0.60)	0.001	0.69 (0.47–1.03)
Model 3	1.00	0.52 (0.22–1.22)	0.17 (0.06–0.49)	0.001	0.60 (0.39–0.93)

^1^All values are odds ratios and 95% confidence intervals. Model 1: Adjusted for age, gender, energy intake. Model 2: More adjustments for physical activity levels, socioeconomic status. Model 3: Further adjustments for total dietary fat intake, plant protein (for animal protein), animal protein (for plant protein) and BMI.

## Discussion

In the present cross-sectional study, we investigated the relation between total and subtypes of dietary protein intake and odds of MUO among Iranian adolescents. Considering IDF criteria, individuals with higher dietary total, plant and animal protein intake were less likely to have MUO after considering all possible covariates. Furthermore, we found a significant inverse relation between dietary total and animal protein intake and odds of being MUO by considering IDF/HOMA-IR criteria. We also found a significant linear association between dietary total and animal protein intake and chance of being MUO. However, no substantial linear relation was observed between dietary plant protein intake and odds of MUO. As far as we know, this was the first study exploring the association between total, plant and animal protein intake and MUO among a sample population of adolescents in the Middle-East.

Adolescence, as a critical development stage in life, needs specific attention. It has been estimated that almost 80% of obese adolescents might be more likely to develop obesity in their adulthood and many health-related comorbidities^41,42^ and mortality^43^. Concomitant existence of obesity with other metabolic abnormalities could augment its health-threatening effects, and could place a great burden on healthcare systems^42^. Therefore, specific attention should be attracted to dietary intakes of children and adolescents that is a primary modifiable factor for obesity and its related diseases. We found that higher protein intakes, from both plant and animal sources and in place of carbohydrate, might be associated with lower possibility of being MUO among Iranian adolescents. It is worth noting that protein sources, especially animal proteins, are often accompanied with some quantities of saturated fats which might have deleterious effects on the adolescents’ health. Therefore, more healthful food choices including fish, nuts, legumes, eggs and low-fat dairy could be a practical approach for decreasing the prevalence of metabolic unhealthy obesity in adolescents.

We found inverse associations between dietary total, plant and animal protein intake and odds of being MUO. As far as we know, studies regarding the relation between dietary protein intake and metabolic phenotypes of obesity have not been widely performed, especially among children and adolescents. In accordance to our findings, a cohort study among 630 Canadian children revealed a marginal inverse relation between higher protein intakes and risk of metabolically unhealthy obesity^44^. However, a cross-sectional study among Latino 10–17 years old adolescents did not found a significant difference in protein intake among individuals with and without metabolic syndrome^45^. A cohort study on 3963 American women did not indicate a significant association between total and animal protein intake and metabolic syndrome incidence^46^. Azemati et al.^47^, in a cross-sectional study among 518 adults in US and Canada, revealed that individuals with higher intakes of total and animal protein were less likely to have metabolic syndrome. In contrast to our findings, a cross-sectional study on 13,485 Korean adults found a positive relation between animal protein intake and metabolic syndrome parameters in men, however, no substantial association was found among women^48^. However, another study found an inverse association between total protein intake and odds of metabolic syndrome in women, but not in men^49^. An earlier meta-analysis revealed that high consumption of total and animal protein intake is related to an increased risk of T2D, however, no significant association was observed between moderate intakes of total and animal protein and T2D risk^50^. Furthermore, a moderate consumption of plant protein was found to be related to 6% reduced risk of diabetes^50^. Another meta-analysis did not found a significant association between total, animal and plant protein intake and risk of hypertension^51^.

Current discrepancies among studies could be explained by several reasons. First, previous studies were mostly conducted among adult populations that have different nutritional requirements from adolescents. Given the lack of studies among adolescents, more prospective studies are required. Second, studies have examined different endpoints that should be cautiously compared with each other. Third, we should keep in mind that dietary intakes in high, middle and low income countries are different, as in low- to middle-income countries, such as Iran, refined carbohydrates are the major sources of energy intake and protein intakes are relatively low^52^. In our study population an equal percentage of energy was contributed to plant and animal proteins. This could explain the inverse association between animal protein intake and metabolic health status, in contrast to some previous investigations. Also, other beneficial nutrients in plant-based sources could neutralize the possible deleterious effects of animal foods. Other reasons for the inconsistencies might be due to differences in study population, approaches of analysis, and measurement tools.

Although the exact mechanism through which protein might improve metabolic status has not been understood, several points should be considered. The inverse association between protein intake and metabolic health is mainly contributed to its substitution for carbohydrate. High carbohydrate intakes, especially from refined sources, could lead to higher serum TG levels and decreased HDL cholesterol, that are main components of metabolic syndrome^53^. Therefore, increasing protein intake in place of carbohydrate could be a beneficial way for improving dyslipidemia. Furthermore, higher protein intake is related to increased secretion of incretin peptides that have an important role in insulin sensitivity^54^. The amino acid content of protein is also important. For instance, branched-chain amino acids, mainly provided by animal sources, could improve metabolic status by their beneficial role in lipogenesis, lipolysis, and glucose metabolism^55^. Moreover, plant-protein sources contain higher amounts of fiber that has been found to be related to improved metabolic status^56^. However, in our study population, red and processed meats that are major sources of animal proteins are not as highly consumed as in Western diets to exert their deleterious effect. Also, previous meta-analyses found little or no association between processed and red meat and cardio-metabolic health, although the certainty of their evidence was low to very low^57,58^.

Our study has several strengths. This is the first study exploring the association of dietary total protein intake and its subtypes with metabolic health status of Iranian overweight and obese adolescents. Furthermore, we used validated questionnaires and blood sample sizes which increased the accuracy of our assessments. Also, several potential confounders were controlled in our analyses. Nevertheless, several limitations should be also acknowledged in our investigation. First, we are not able to infer a causal relation due to the cross-sectional nature of this study. Therefore, prospective studies are warranted to detect the relation between protein intake and metabolic health status. Second, misclassification of participants was inevitable because of the possibility of measurement errors of FFQ. Third, despite controlling several covariates, some residual confounders might still affect the relations. Finally, we did not collect any data on mood and well-being, and it is suggested for the future studies to examine mood status of adolescents besides assessing their metabolic health. Our study was performed among a sample of overweight and obese adolescents in a low to middle income country. Given the differences in dietary intakes of countries, especially in case of macronutrients intake, the findings should be cautiously extrapolated to other populations. Further well-designed studies among different nations are required.

In conclusion, we found inverse associations between total, plant, and animal protein and odds of being MUO among adolescents. Therefore, reducing carbohydrate in favor of protein intake could be a worthwhile approach for public health strategies in developing countries. Further studies are needed to confirm our results.

## Data Availability

The data that support the findings of this study are available from the corresponding author upon reasonable request.

## Abbreviations

FFQ: Food frequency questionnaire
OR: Odds ratios
95% CI: 95% Confidence interval
BMI: Body mass index
MHO: Metabolically healthy obesity
MUO: Metabolically unhealthy obesity
IDF: International Diabetes Federation
HOMA-IR: Homeostasis Model Assessment Insulin Resistance
PAQ-A: Physical Activity Questionnaire for Adolescents
SES: Socioeconomic status
WC: Waist circumference
SBP: Systolic blood pressure
DBP: Diastolic blood pressure
TG: Triglycerides
HDL-c: High density lipoprotein cholesterol
FBG: Fasting blood glucose
T2D: Type 2 diabetes
ANOVA: Analysis of variance
ANCOVA: Analysis of covariance
SPSS: Statistical package for the social sciences
